# An insight into the prokaryotic diversity from a polymetallic nodule-rich region in the Central Indian Ocean Basin using next generation sequencing approach

**DOI:** 10.3389/fmicb.2024.1295149

**Published:** 2024-03-18

**Authors:** Shruti Shah, Samir R. Damare, Maria Brenda Luzia Mascarenhas-Pereira, Jayesh Patil, Sneha Parab, Sushil Nair, Arpita Ghosh

**Affiliations:** ^1^Biological Oceanography Division, CSIR-National Institute of Oceanography, Panaji, India; ^2^School of Earth, Ocean, and Atmospheric Sciences, Goa University, Taleigão, India; ^3^Geological Oceanography Division, CSIR-National Institute of Oceanography, Panaji, India; ^4^Eurofins India Pvt. Limited, Bengaluru, India

**Keywords:** abyss, Central Indian Basin, eDNA, oligotrophic waters, prokaryotic diversity, pelagic zone, polymetallic nodules

## Abstract

Deep sea is a vast, dark, and difficult-to-access terrain and is now looked upon as a unique niche harboring diverse microorganism. We used a metataxonomic approach to decipher the microbial diversity present in the water column (surface to near bottom), water overlaying the sediments, and the deep-sea sediments (up to 35 cm) from the Indian Contract Region (ICR) in the Central Indian Ocean Basin (CIOB). Samples were collected from #IRZ (Impact Reference Zone), #PRZ (Potential Reference Zone), and #BC20 (Control site, outside potential mining area) with an average water depth of 5,200 m. 16S rRNA (V3–V4 region) amplicon sequencing on the MiSeq platform resulted in 942,851 ASVs across 65 water and sediment samples. Higher prokaryotic diversity was observed below 200 m in the water column to the seafloor. Proteobacteria was the most dominant bacterial phylum among all the water samples while Firmicutes, Actinobacteria and, Bacteroidota dominated the sediments. Sediment (below 10 cm) was co-dominated by Firmicutes. Thermoplasmata was the dominant archaeal group in the water column while Crenarchaeota was in the sediments. BC20 was less diverse than IRZ and PRZ. Deep Sea microorganisms could play a vital role in the mineralization processes, nutrient cycling, and also different biogeochemical cycles.

## Introduction

Prokaryotes are the most abundant (1 × 10^29^ cells mL^−1^) group of microorganisms in the largest ecosystem on Earth, the Oceans (Ferrera et al., 2015). They account for 70 to 75% of the total biomass in the surface and deeper waters (Jing et al., 2013). They are vital components of the planktonic lifeforms and represent a huge fraction of the genetic and metabolic diversity of life forms on Earth (Fuhrman et al., 1993; Oren, 2004). They play a major role in biogeochemical cycles, nutrient cycling, ecosystem functioning and marine food chains, and global climate change by mediating different chemical transformations in various biochemical reactions (Azam and Malfatti, 2007; Falkowski et al., 2008). Of all the microorganisms present in the marine environment only about 0.001 to 15% can be isolated and cultured (Hongxiang et al., 2008).

The Central Indian Ocean Basin (CIOB) (0°–25°S and 70°–90°E) is an oligotrophic region and is poorly studied for its microbial diversity compared to other oceans (Wei et al., 2019). There are a few reports on the isolation and cultivation of various microbial species from the Indian Ocean (Wang et al., 2016). The abyssal plains (3,000 to 6,000 m) of the CIOB possess an average temperature and pressure of about 2°C and 50 MPa with a total organic carbon (TOC) content of less than 0.01% (Das et al., 2010). This region has been previously explored for the nature, distribution, and abundance of polymetallic nodules from the late 1980s (Sharma and Nath, 2000). The CIOB has been explored extensively for sediment characteristics, hydrothermal activity, tectonic features, seamounts, etc. It was found that siliceous sediments and pelagic red clay dominate north and south of CIOB, respectively (Naik et al., 2016). Microbial and geological studies at CIOB have suggested the importance of microorganisms for mineral exploration and as indicators in biogeochemical processes and their role and response to artificially simulated disturbance (Naik et al., 2016).

Next generation sequencing (NGS) techniques have deepened our understanding of microbial diversity, evolution, and their functional potential (Sunagawa et al., 2015; Xie et al., 2022). There have been a few reports of microbial diversity studies from the Indian Ocean. Barnes et al. (2021) in their review provided insights into the different NGS techniques used to decipher the microbial diversity from different regions of the Indian Ocean. Wang et al. (2016) reported the abundance and distribution of bacteria in the water column of the Eastern Indian Ocean where Cyanobacteria and Actinobacteria were dominating the upper ocean while Alphaproteobacteria were in the deeper waters. Another study reported the occurrence of the *Prochlorococcus*, and *Synechococcus* accounting for 90% of the total cyanobacterial reads (Díez et al., 2016). A stable microbial community dominated majorly by SAR11, *Prochlorococcus* and *Synechococcus* was observed in the surface waters of the Northern Indian Ocean (Xie et al., 2022). Another study reported a higher abundance of the organisms belonging to the Epsilon-proteobacteria group from the Edmon hydrothermal vent on Central Indian Ridge (Hoek et al., 2003). A higher abundance of Proteobacteria group has been reported from the interior and exterior carbonate sediments of the Southwest Indian Ridge (Li et al., 2014).

Microorganisms are crucial components of any ecosystem. They are robust and adaptive to varying environmental conditions. Hence it is interesting to explore the microbial diversity present in these waters which will facilitate our understanding of the different processes in the marine environment. This study aims to decipher the prokaryotic diversity from the water column and the sediments of a polymetallic nodule-rich region in the CIOB using the next generation sequencing technique.

## Methodology

### Sampling

Water samples (10 L each) were collected from the three locations #BC20 (Control Site, outside the proposed trial mining area) (12.4° S–75.33° E), #PRZ (Potential Reference Zone) (12.56° S–74.41° E) and #IRZ (Impact Reference Zone) (13.4° S–75.33° E) from the CIOB in July 2019 on-board RV Sindhu Sadhana (cruise #SSD062). IRZ location is the proposed mining area, while PRZ is a similar area to the IRZ in terms of topography and nodule abundance in the Indian Contract Region. BC20, is a topographically similar area, having very few scattered polymetallic nodules, treated as a control location. This sampling was a part of the Environmental Impact Assessment (PMN-EIA) program funded by MoES, Govt. of India for potential polymetallic nodule mining in the CIOB in the Indian Contract Region. Water samples were collected from depths of 30 m, chlorophyll maxima (C_max_), 200 m, 600 m, 2,000 m, 3,500 m, and near the bottom with the help of Niskin bottles attached to the Conductivity-Temperature-Depth (CTD) rosette of 24 bottles (SeaBird, United States), each with 10 L capacity. Samples were filtered directly using a 0.22 μm cartridge filter (Sterivex filter) (Millipore, United States). C_max_ for #BC20 and #PRZ was at 90 m, while for #IRZ it was 84 m. Similarly, the near bottom sampled depth for BC20 was 5,200 m, while for IRZ, it was 5,130 m. Water samples were coded as BW (BW1–BW7), IW (IW1–IW7), and PW (PW1–PW6).

Sediment samples were collected using multicore (Maxi Multiple Corer types of Bowers & Connelly Corer, United States) from #BC20 (BS), #IRZ (IS), and #PRZ (PS). The core obtained at the stations was 35 cm bsf long. Water overlaying sediment from the cores was collected (approx. 10 L each) from all three stations, i.e., BC20 (BSW), IRZ (ISW), and PRZ (PSW). Each sediment core was sectioned into sub-sections as 0–0.5, 0.5–1, 1–1.5, 1.5–2, 2–3, 3–4, 4–6, 6–8, 8–10, 10–15, 15–20, 20–25, 25–30, 30–35 cm bsf. These subsections were coded as per the locations and ascending order (For BS: BS1–BS14; IS: IS1–IS14 and PS: PS1–PS14). Each sub-section was put into sterile bags and stored onboard at −20°C. These samples were then transported to the laboratory under frozen conditions. In the laboratory, the sub-sectioned sediment samples were freeze-dried in a freeze-dryer (Labconco, United States), and the dried samples were transferred to sterile tubes until further use.

### Measurements of the physico-chemical parameters

Physico-chemical parameters such as temperature, dissolved oxygen (DO), salinity, suspended particulate matter (SPM), pH, phosphate, nitrate, and nitrite were measured for the water samples. While nitrate, nitrite, phosphate, total organic carbon (TOC), total nitrogen (TN) and, labile organic matter (LOM) was measured for the sediments using standard methods.

For metal analysis, cores were sub-sampled immediately with a 2 cm interval. The samples were frozen and stored onboard. In the lab, the samples were completely dried and powdered into very fine particles using mortar and pestle. About 50 mg of bulk samples were accurately weighed in a pre-acid cleaned PTFE vials and digested using an acid mixture (7:3:1; HF:HNO_3_:HClO_4_), followed with aqua-regia (1:3; HNO_3_:HCl) treatment. After digestion, the acids were completely allowed to dry and re-dissolved in a mild HNO_3_ for trace metals and major elements analysis. Abundances of trace elements were measured using HR-ICP-MS (Attom ES, Nu Instruments, United Kingdom), whereas major elements (e.g., Na, K, Mg, Fe, and Mn) in these solutions were measured using an ICP-OES (Perkin Elmer, United States).

### DNA extraction and amplicon sequencing

DNA extraction was performed onboard for the water samples to avoid the freeze–thaw of the samples. Post filtration, the Sterivex filter cartridges were cracked open and the filter paper was chipped into small pieces and resuspended in the lysis buffer of the E.Z.N.A.^®^ Soil DNA kit (Omega Bio-tek, United States). DNA extraction was performed as per the manufacturer’s instructions. For sediment samples, 500 mg of dried sediment samples were used for DNA extraction. Extracted DNA samples were dried in the vacuum concentrator (Eppendorf, Germany) and were outsourced for amplicon sequencing (V3–V4 region of 16S rRNA).

The amplicon library was prepared using Nextera XT Index Kit (Illumina Inc.) according to the 16S Metagenomic Sequencing Library preparation protocol (Part # 15044223 Rev. B). Bacteria-specific region primers were designed and synthesized at Eurofins Lab for amplification. The primer sequences were *16S rRNA F* GCCTACGGGNGGCWGCAG and *16S rRNA R* ACTACHVGGGTATCTAATCC, respectively. The QC passed libraries were sequenced on the Illumina MiSeq platform with 2 × 300 bp chemistry.

### Bioinformatics and statistical analyses of the amplicon-sequenced data

The raw paired-end reads were subjected to quality check using Fast QC tool (FastQC, n.d.) and trimming by Trimmomatic v 0.38 (Bolger et al., 2014) respectively, resulting in high-quality clean reads. Clean high-quality reads were further analyzed in QIIME2 2022.2 pipeline (Bolyen et al., 2019) using DADA2 Package (Callahan et al., 2016) to obtain Amplicon Sequence Variants (ASVs). Clustering of the obtained ASVs into their respective groups was done using SILVA 138 SSU reference database (Quast et al., 2013). Rarefied ASVs were used for further downstream diversity analyses. The raw paired-end reads generated from the Illumina MiSeq platform were submitted to NCBI Sequence read Archive (SRA) with BioProject ID PRJNA668687.

Microbiome and Phyloseq packages in RStudio (RStudio Team, 2020) with R 4.1.0 package were used to obtain the alpha diversity indices such as Shannon, Simpson’s richness, observed species, and Pielou’s evenness (RStudio Team, 2020). Correlation plots were constructed in PAST 4.07b (Hammer et al., 2001) to assess the correlation between microbial groups and the physicochemical parameters. A vertical comparison of the data obtained from the samples of the water column and the sediments was performed to evaluate the vertical prokaryotic distribution. Also, data obtained from the samples were compared to assess the differences between prokaryotic diversity across these three locations. Similarity percentage analysis (SIMPER—R vegan function) was performed to assess the contribution of the taxonomic group contributing maximum towards the differences in the abundances between the samples or sample groups. For this, we divided the samples into three groups, namely, water, sediment, and overlaying water samples for ease of understanding and comparison. For water samples, a comparison of all the water depths was made against the topmost one (30 m) to understand how the taxa change with depth. Similarly, for sediment samples, all the sediment samples were compared against the topmost sediment sub-section (0–0.5 cm) to assess the change in the abundance of the taxa with depth. Also, two additional comparisons were made: water samples of BC20 station to IRZ and PRZ, respectively, to assess the dissimilarity in the water column depth across the three locations. Similarly, all the water, sediment, and overlaying water samples of BC20 were compared against the IRZ and PRZ, respectively, to assess the overall dissimilarity across the three locations.

## Results

### 16S microbiome profiling and alpha diversity

A total of 65 samples were used in this study, 14 water, 42 sediment sub-sections and three water samples overlaying sediment. 16S Microbiome profiling (V3–V4 region of the 16S rRNA gene sequences) of these 65 samples yielded a total of 11,018,334 reads. The rarefaction curve of the samples almost bent to the saturation plateau for most samples indicating that sequencing depth was sufficient to obtain the necessary information on the microbial diversity present in the samples under consideration in this study.

Alpha diversity depicted higher microbial diversity in the bottom waters, i.e., below 1,000 m (Table 1). At station BC20, the highest alpha diversity was found to be BW3 (200 m), followed by BW5 (2000 m) and BW6 (3,500 m). At station IRZ, the highest alpha diversity was found to be at ISW (overlaying water) followed by IW6 (3,500 m), IW7 (5,130 m) and IW5 (2,000 m) respectively. At station PRZ, the highest alpha diversity was found at PW6 (3,500 m) and PSW (overlaying water). Briefly, 837 observed species and a Shannon index of 6.25 were noted at ISW, which was the maximum amongst all the 65 samples. This was followed by 816 observed species and a 6.32 Shannon index at IW6 and 790 observed species and a 6.21 Shannon index at IW6, respectively.

**Table 1:** Alpha diversity of all the 65 samples across the three locations.

Samples	Observed species	Shannon	Simpson	Pielou’s evenness
BW1	69	3.496	0.964	0.826
BW2	65	3.056	0.936	0.732
BW3	519	5.696	0.995	0.911
BW4	263	4.893	0.990	0.878
BW5	460	5.508	0.994	0.898
BW6	307	5.169	0.993	0.903
BW7	283	4.941	0.990	0.875
BSW	117	2.805	0.872	0.589
BS1	21	2.444	0.877	0.803
BS2	48	3.465	0.962	0.895
BS3	15	2.146	0.858	0.792
BS4	13	2.041	0.843	0.796
BS5	14	1.644	0.759	0.623
BS6	22	2.722	0.924	0.881
BS7	12	1.975	0.838	0.795
BS8	8	1.632	0.770	0.785
BS9	7	1.628	0.774	0.837
BS10	21	2.323	0.881	0.763
BS11	9	1.758	0.800	0.800
BS12	18	2.165	0.860	0.749
BS13	11	1.627	0.721	0.678
BS14	44	3.017	0.935	0.797
IW1	80	3.602	0.964	0.822
IW2	16	2.179	0.865	0.786
IW3	40	2.702	0.910	0.732
IW4	249	4.943	0.990	0.896
IW5	586	5.793	0.995	0.909
IW6	816	6.317	0.997	0.942
IW7	790	6.206	0.996	0.930
ISW	837	6.247	0.997	0.928
IS1	170	4.649	0.988	0.905
IS2	85	3.682	0.966	0.829
IS3	124	4.105	0.978	0.852
IS4	87	3.607	0.964	0.808
IS5	126	4.228	0.981	0.874
IS6	126	3.984	0.975	0.824
IS7	111	3.967	0.975	0.842
IS8	37	2.630	0.908	0.728
IS9	26	2.719	0.925	0.835
IS10	59	3.284	0.950	0.806
IS11	76	3.519	0.957	0.813
IS12	24	2.535	0.893	0.798
IS13	36	2.867	0.928	0.800
IS14	46	3.286	0.954	0.858
PW1	29	2.806	0.925	0.833
PW2	58	3.320	0.951	0.818
PW3	37	2.865	0.929	0.793
PW4	27	2.684	0.913	0.814
PW5	150	4.359	0.982	0.870
PW6	524	5.830	0.996	0.931
PSW	468	5.563	0.994	0.905
PS1	141	4.269	0.981	0.863
PS2	161	4.209	0.979	0.828
PS3	121	3.982	0.975	0.830
PS4	166	4.427	0.984	0.866
PS5	143	4.465	0.984	0.900
PS6	118	3.968	0.974	0.832
PS7	194	4.874	0.990	0.925
PS8	177	4.626	0.987	0.894
PS9	37	2.996	0.928	0.830
PS10	29	2.811	0.932	0.835
PS11	33	2.969	0.934	0.849
PS12	33	2.794	0.922	0.799
PS13	22	2.453	0.898	0.794
PS14	28	2.829	0.925	0.849

### Taxonomic abundance of the prokaryotic community

Of the total 942,851 ASVs obtained, 884,780 ASVs categorized as Bacteria (93.84%), 57,672 for Archaea (6.12%) and 399 ASVs remained Unassigned (0.04%). These ASVs at the respective locations were taxonomically categorized into their respective groups and they clustered into 53 different phyla, 136 classes, 343 orders, 546 families, 954 genera and 1,565 species. At, BC20, the ASVs categorized into a total of 37 phyla, 73 classes, 157 orders, 247 families, 365 genera and 541 species. At IRZ, the ASVs categorized into a total of 51 phyla, 126 classes, 236 orders, 466 families, 782 genera and 1,193 species. At PRZ, the ASVs categorized into a total of 44 phyla, 99 classes, 212 orders, 363 families, 522 genera and 746 species.

### Prokaryotic diversity across the locations

**Figure 1:**
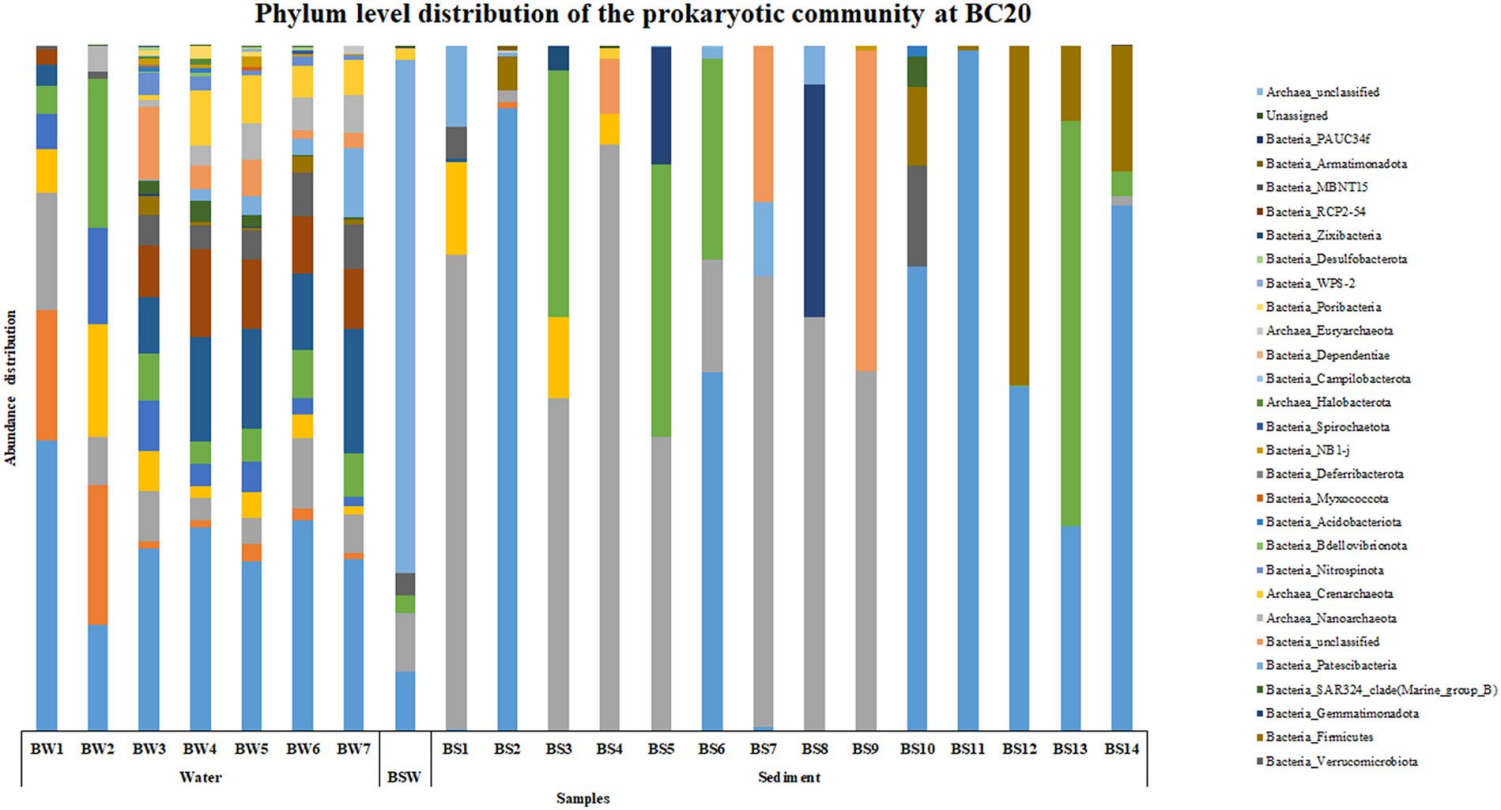
Phylum level distribution of the prokaryotic community at BC20.

An interesting trend was observed in the abundance and distribution of the microbial groups across the water and sediment samples within each location and across locations. At BC20, Proteobacteria was the most abundant phylum throughout the water column, while in sediments, at BS1 (0–0.5 cm), BS3 (1–1.5 cm), BS4 (1.5–2 cm), BS5 (2–3 cm), BS7 (4–6 cm), BS8 (6–8 cm) and BS9 (8–10 cm) phylum Bacteroidota was the most abundant microbial group (Figure 1). Alphaproteobacteria was the abundant class at BW1 (30 m), BW4 (600 m), BW5 (2,000 m) and BW6 (3,500 m) in the water column. Parcubacteria was the most abundant class at PSW (overlaying water). In the sediments, BS1 (0–0.5 cm), BS3 (1–1.5 cm), BS4 (1.5–2 cm), BS5 (2–3 cm), BS7 (4–6 cm) and BS9 (8–10 cm), Bacteroidia was the most abundant class, while at BS12 (20–25 cm), BS13 (25–30 cm) and BS14 (30–35 cm), Bacilli, Actinobacteria and Gammaproteobacteria were the abundant classes, respectively, (Supplementary Figure S1). The abundance of *Candidatus_Kaiserbacteria*, *Nocardioides*, *Roseibacillus*, and *Erythrobacter* increased with water column depth. Genera such as *Marinimicrobia*_(SAR406_clade), SAR11_clade, SAR202_clade, and *Rhodopirellula* were abundant throughout the water column. However, the maximum number of ASVs was observed in the mid, i.e., from 200 to 3,500 m in the water column. In the sediments, *Sphingomonas*, *Aquibacter*, *Bacillus*, *Ulvibacter*, *Reyranella*, and *Alcanivorax* were the most abundant genera across all the subsections.

**Figure 2:**
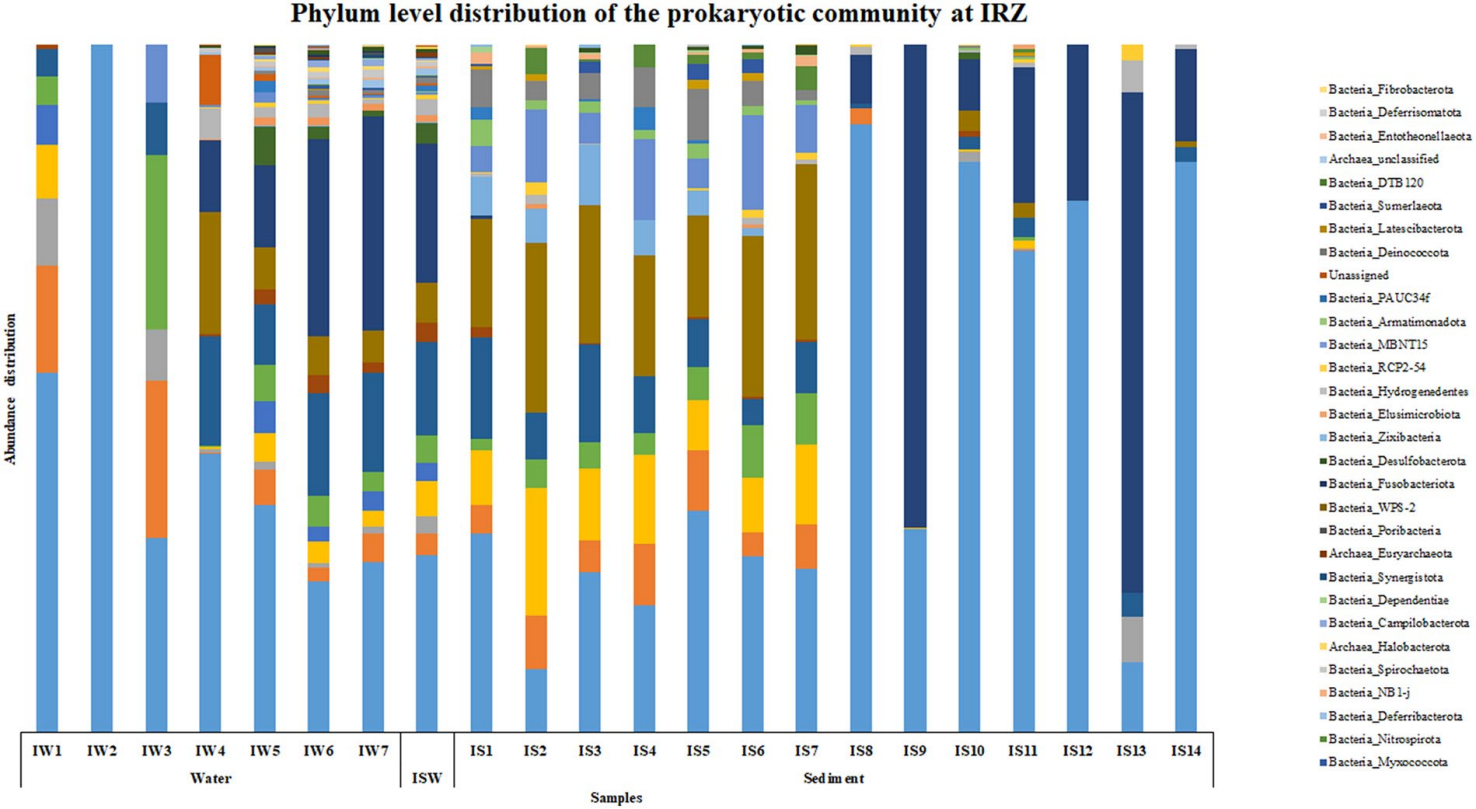
Phylum level distribution of the prokaryotic community at IRZ.

At IRZ, phylum Proteobacteria was the most abundant microbial group up to IW5 (2000 m) in the water column and at ISW, while Firmicutes was the most abundant phylum at IW6 (3,500 m) and IW7 (5,130 m) respectively. In the sediment samples of IRZ, Proteobacteria was the most abundant phylum except at IS1 (0–0.5 cm), IS7 (4–6 cm), IS9 (8–10 cm) and IS13 (25–30 cm) where phylum Actinobacteria was the most abundant microbial group (Figure 2). At the class level, Alphaproteobacteria was the most abundant group across most of the samples at IRZ. Below IW5 (2000 m), the abundance shifted from Alphaproteobacteria to Gammaproteobacteria and at IS1 (0–0.5 cm), IS5 (2–3 cm) and IS8 (6–8 cm) respectively. IS2 (0.5–1 cm) to IS4 (1.5–2 cm), IS6 (3–4 cm) and IS7 (4–6 cm) exhibited a higher abundance of Acidimicrobiia group (Supplementary Figure S2). *Marinimicrobia*_(SAR406_clade), SAR11_clade, SAR202_clade, *Marinobacter*, and AEGEAN-169_marine_group were the abundant genera present throughout the water column with higher abundance distributed in 200 to 3,500 m in the water column. The majority of the genera detected at IRZ either showed an increase or decrease in abundance with depth. Genera such as *Lactobacillus*, *Prevotella*, *Pseudomonas*, *Bacteroides*, *Odoribacter*, *Acinetobacter*, *Pseudoalteromonas*, and SAR324_clade (Marine_group_B) showed an increase in abundance with depth. While *Alcanivorax*, *Nocardioides*, *Peredibacter*, *Nitrospina*, *Muricauda*, *Hyphomonas* and *Bdellovibrio* were the genera whose abundance decreased with depth. In sediments, the majority of the genera showed higher abundance in the upper subsections, i.e., up to 6 cm. These include *Woeseia*, Dadabacteriales, Urania-1B-19_marine_sediment_group, *Aquibacter*, *Nitrospira*, *Nitrosomonas*, *Candidatus_Nitrosopumilus*, Sva0996_marine_group, Marine_Benthic_Group_A, and Nitrosopumilaceae, respectively. Genera that showed higher abundance below 6 cm were *Jeotgalibacillus*, *Sphingomonas*, *Reyranella*, and *Turicibacter*, respectively.

**Figure 3:**
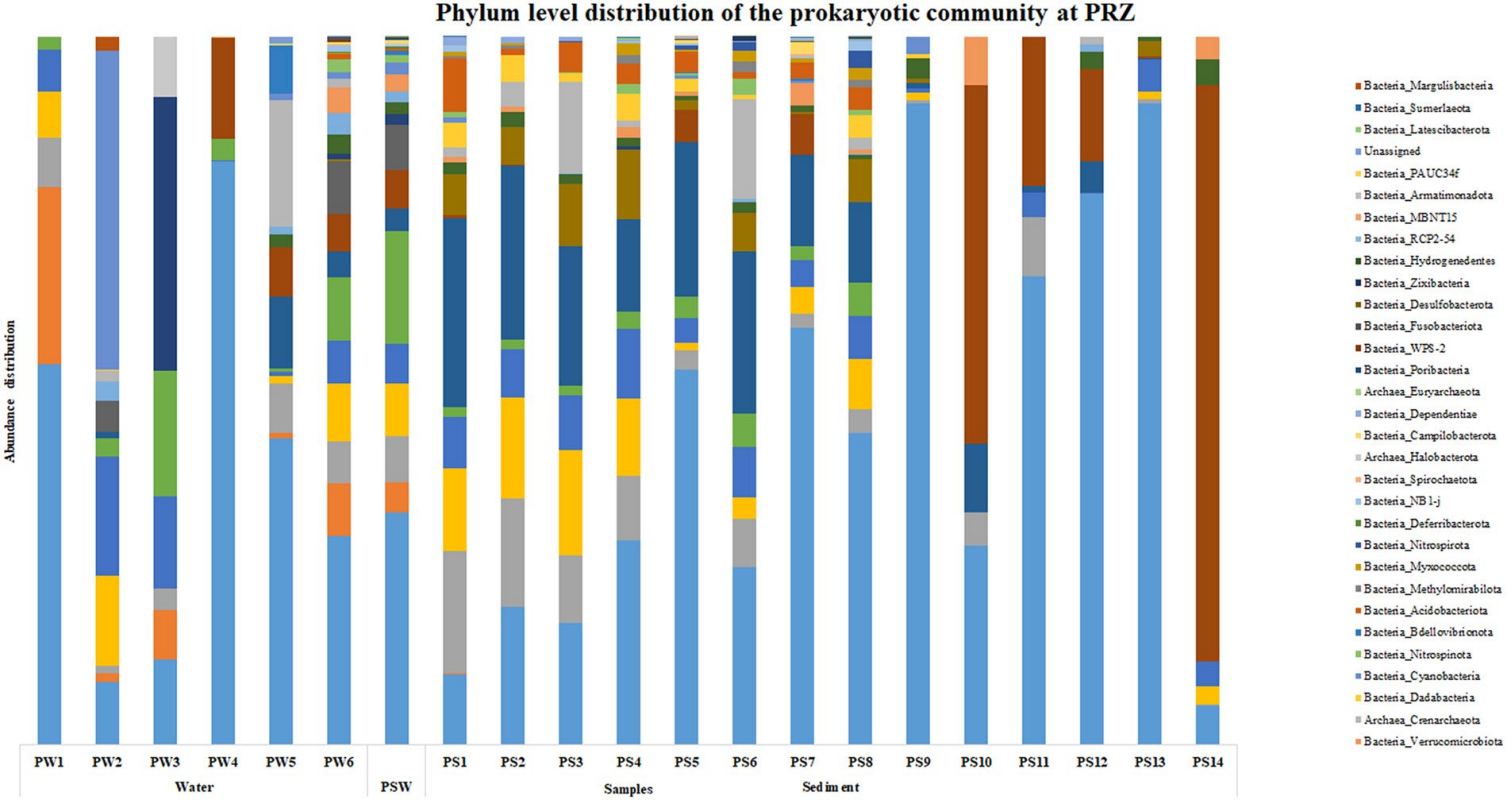
Phylum level distribution of the prokaryotic community at PRZ.

At PRZ, phylum Proteobacteria was the most abundant microbial group in the water column except at PW2 (90 m) where Cyanobacteria was the most abundant phylum. SAR324 clade (Marine group B) was the most abundant phylum at PW3 (200 m). In the sediments, Actinobacteria was the most abundant phylum in PS1 (0–0.5 cm), PS2 (0.5–1 cm), PS3 (1–1.5 cm) respectively. PS10 (10–15 cm), and PS14 (30–35 cm) exhibited a higher abundance of Firmicutes group (Figure 3). At the class level, Alphaproteobacteria was found to be abundant at PW1 (30 m), PW5 (2000 m), PS7 (4–6 cm), and PS10 (10–15 cm) respectively. Cyanobacteria and SAR324 clade (Marine group B) were the most abundant classes at PW2 (90 m) and PW3 (200 m) respectively. Gammaproteobacteria was found to be highly abundant at PW4 (600 m), PS4 (1.5–2 cm) to PS9 (8–10 cm) and PS11 (15–20 cm) to PS13 (25–30 cm) respectively. PS1 (0–0.5 cm) to PS3 (1–1.5 cm), and PS6 (3–4 cm) exhibited an abundance of the Acidimicrobiia group. A higher abundance of class Bacilli were observed at PS10 (10–15 cm), and PS14 (30–35 cm) respectively (Supplementary Figure S3). Genera such as *Marinimicrobia*_(SAR406_clade), SAR202_clade, AEGEAN-169_marine_group and SAR11_clade were abundant throughout the water column. While SAR86_clade, *Lactobacillus*, *Woesearchaeales*, *Marinobacter*, and *Alcanivorax* showed an increase in abundance with water column depth. In the sediments, a higher abundance in genera was detected in the top 10 cm. These include *Aquibacter*, Urania-1B-19_marine_sediment_group, *Nitrospina*, *Nitrospira*, Marine_Benthic_Group_A and Candidatus_*Nitrosopumilus*. *Jeotgalibacillus* and *Shewanella* were detected only below 10 cm in the sediment samples.

Based on the SIMPER analysis, at BC20, the taxa that contributed to the dissimilarity in the water column depth when compared with the 30 m (topmost sample) were *Nocardoides*, *Alteromonas*, and *Alcanivorax*. In the sediments, *Alcanivorax*, *Reyranella*, *Sphingomonas*, *Bacillus*, and *Candidatus Nitrosopumilus* were the taxa that contributed to the dissimilarity when compared to the 0–0.5 cm (topmost) sub-section. At IRZ, the taxa that contributed to the dissimilarity in the water column depth when compared with the 30 m (topmost sample) were *Lactobacillus*, *Marinobacter*, *Alteromonas*, *Pseudomonas*, and *Pseudoalteromonas*. While in the sediments, *Jeotgalibacillus*, *Sphingomonas*, *Reyranella*, *Woeseia*, and *Turicibacter* were the taxa that contributed to the dissimilarity when compared to the 0–0.5 cm (topmost) sub-section. At PRZ, *Lactobacillus*, *Alteromonas*, and *Bacillus* were the taxa that contributed to the dissimilarity in the water column depth when compared with the 30 m (topmost sample). While in the sediments, the taxa that contributed to the dissimilarity when compared to 0–0.5 cm (topmost) sub-section were *Jeotgalbacillus*, *Shewanella*, *Aquibacter*, *Nitrospira* and *Woeseia*. On comparing the water samples of IRZ and PRZ against the BC20, the taxa that contributed to the dissimilarity across the sample groups were *Alteromonas*, *Pseudomonas*, *Pseudoalteromonas*, *Nocardioides*, *Alcanivorax*, and *Lactobacillus*, respectively. While comparing IRZ and PRZ against the BC20 station, the taxa that contributed to the dissimilarity across the sample groups were *Sphingomonas*, *Shewanella*, *Bacillus*, *Alteromonas*, and *Pseudomonas*, respectively.

### Correlation of prokaryotic diversity to the physicochemical parameters

**Figure 4:**
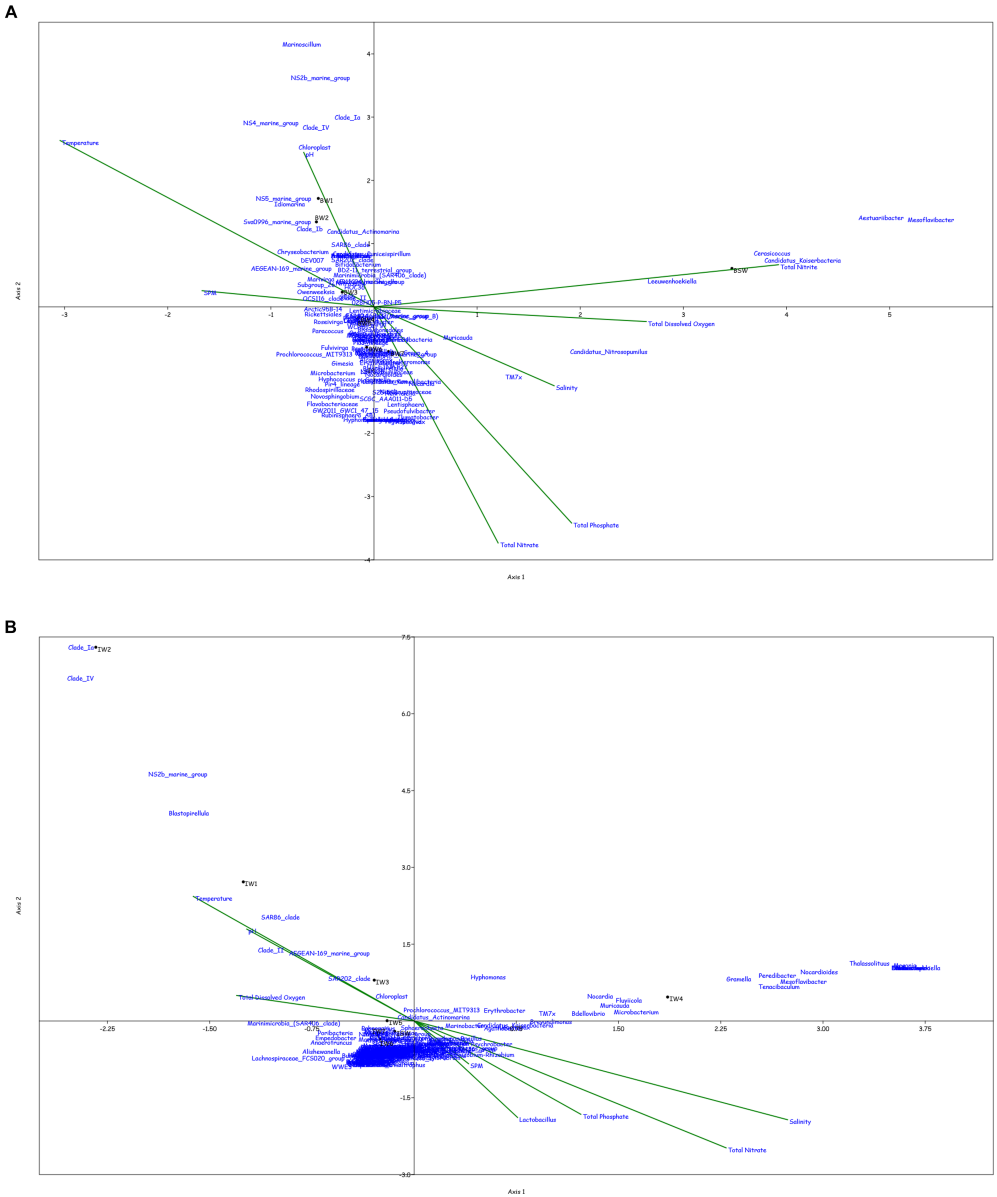

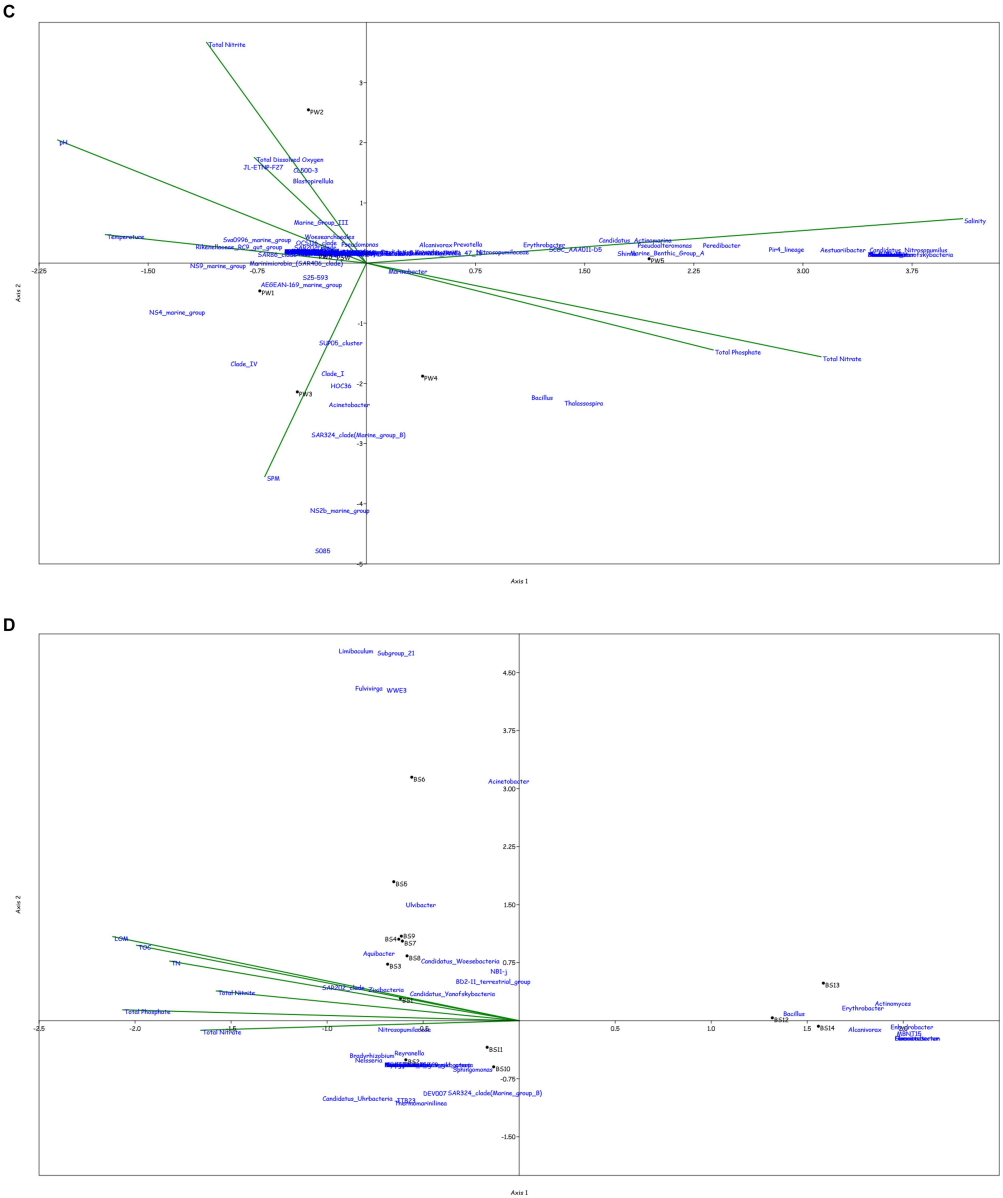

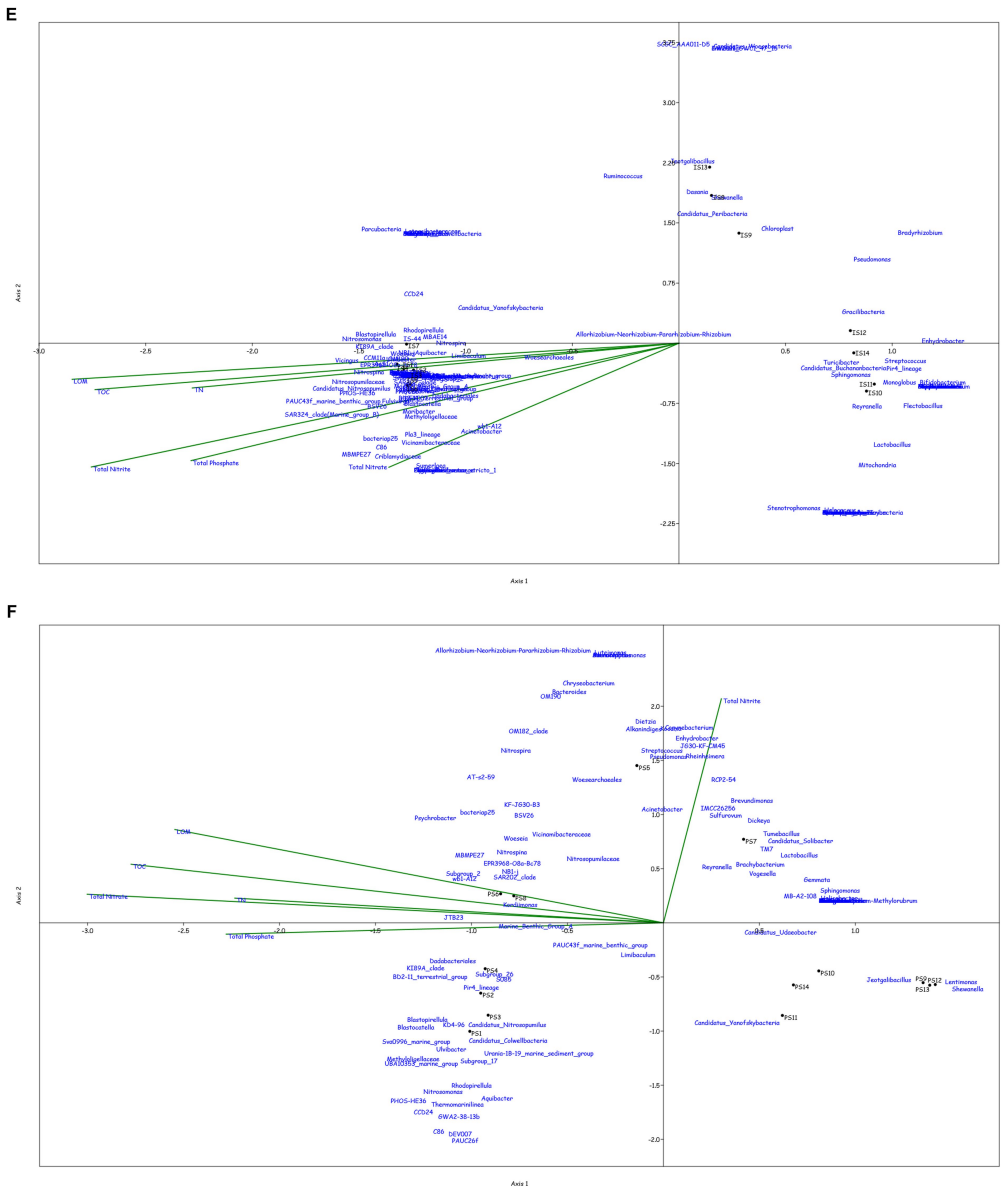
Canonical Correspondence analysis showing effect of physico-chemical parameters on the microbial community at genera level in the water column at **(A)** BC20, **(B)** IRZ, **(C)** PRZ and in the sediments at **(D)** BC20, **(E)** IRZ and **(F)** PRZ, respectively.

Canonical correspondence analysis was performed at the genus level to assess the correlation between the physicochemical characteristics of the sampled location and the prokaryotic diversity obtained by 16S amplicon sequencing. Only the top 0.1% genera were taken into consideration for the plot, excluding the uncultured or unclassified genera for the ease of representation (Figure 4).

At BC20, in the water column (Figure 4A), microbial genera present in the upper depths were found to be greatly influenced by temperature while the organisms from the lower depths were found to be influenced by phosphates and nitrates. *Marinimicrobia* (SAR406_clade), *Idiomarina*, *Candidatus_Actinomarina*, NS4_marine_group, and SAR86_clade microbial groups were positively correlated with temperature, pH, and SPM, while negatively correlated with nitrate, phosphate, and dissolved oxygen (DO). *Candidatus_Nitrosopumilus*, *Muricauda*, *Lentisphaera*, and TM7x were positively correlated with the dissolved oxygen and negatively correlated with temperature and SPM. In the sediments (Figure 4D), microbial groups present in the upper subsections (above 10 cm) were greatly influenced by the nutrients, TOC, TN, and LOM. *Aquibacter*, *Candidatus_Yanofskybacteria*, *Zixibacteria*, SAR202_clade, and Nitrosopumilaceae were positively correlated with the TOC, TN, nutrients, and LOM, while *Bacillus*, *Erythrobacter*, *Actinomyces*, *Enhydrobacter*, and *Alcanivorax* were negatively correlated with nutrients, LOM, TOC, and TN.

At IRZ, in the water column (Figure 4B), the upper depths of the water column (up to 200 m) were found to be influenced by temperature and DO while the lower depths IW5, IW6, IW7 and ISW were seen to be influenced by nitrate and phosphate. SAR86_clade, SAR202_clade, *Blastopirellula*, *Prochlorococcus*, and AEGEAN-169_marine_group were positively correlated with temperature and Do while *Thalassolituus*, *Gramella*, *Peredibacter*, and *Mesoflavibacter* were negatively correlated. Nitrosopumilaceae, *Nitrospina*, *Odoribacter*, and *Bacillus* were positively correlated with nutrients such as phosphates, and nitrates. In the sediments (Figure 4E), IS1 to IS9 exhibited a greater influence by nutrients, TOC, TN, and LOM. *Nitrospina*, *Nitrosopumilaceae*, SAR324_clade (Marine_group_B), *Acinetobacter*, *Maribacter*, and *Ulvibacter* are some of the genera that demonstrated positively correlation with the nutrients, TOC, TN and LOM, while *Sphingomonas*, *Reyranella*, *Streptococcus*, and *Turicibacter* were negatively correlated.

At PRZ, in the water column (Figure 4C), the lower depths (PS6 and PSW) were found to be influenced by DO, temperature, and nitrite. Genera such as *Blastopirellula*, SAR86_clade, CL500-3 (family- Phycisphaeraceae) and SAR202_clade positively correlated to nitrite and DO while negatively correlated to phosphate, and nitrate. While *Bacillus* and *Thalassospira* positively correlated to nitrate and phosphate. In the sediments (Figure 4F), majority of the subsections were least influenced by nutrients, TOC, TN, and LOM. Microbial groups such as Marine_Benthic_Group_A, *Kordiimonas*, SAR202_clade, Nitrosopumilaceae, *Nitrospina*, and *Woeseia* positively correlated with LOM, TOC, TN, and nitrate while negatively correlated with nitrite. *Acinetobacter*, *Reyranella*, *Brevundimonas*, and *Sulfurovum* positively correlated with nitrite. Overall, it was found that most of the organisms with higher abundance showed a positive correlation with physicochemical parameters.

Metal analysis of the major elements such as Na, K, Mg, Fe, and Mn (vital for the cell functioning) indicated that their concentrations were found to be slightly higher at PRZ and IRZ where the polymetallic nodules were present than BC20 (control site- no polymetallic nodules). On comparing the three sites, Na was observed to be at a higher concentration in the upper subsections and its concentration decreased with depth. Similar trend was observed for all the other elements at BC20. The concentration of Fe and Mg was found to increase with depth at PRZ and IRZ. While the concentration of K and Mn did not show much variation with depth (Supplementary Figure S4). Metal analysis of the trace elements such as V, Cr, Co, Ni, Cu, and Zn indicated that their concentrations were higher at PRZ and IRZ where the polymetallic nodules were present than BC20. On comparing the three sites for the trace metals analyzed, the elements from their higher to lower concentrations were Ni > Cu > Zn > Co > V > Cr. There was not much variation with depth observed for the trace metals across the three locations (Supplementary Figure S5).

## Discussion

The advancements in the NGS technologies and use of culture-independent approaches have improved our knowledge and provided deeper insights into the community structure and functioning (Smedile et al., 2014). The use of culture-independent methods has also facilitated deciphering the abundance and diversity of the oligotrophic waters which is quite high and their role in nutrient cycling and as drivers of major biogeochemical cycles (Rusch et al., 2007; Sunagawa et al., 2015). The depths of the Indian Ocean are comparatively underexplored, unlike the Pacific and the Atlantic Ocean (Barnes et al., 2021). This study reports the microbial diversity present in the water column as well as sediments of the polymetallic nodule mining region (PMN region) of the CIOB.

The locations sampled in this study are the potential polymetallic nodule mining areas, a part of deep-sea mining in the Indian Ocean, initiated by the Government of India. 16S Microbiome Profiling across the three locations indicated that the microbial diversity across the locations is quite similar, especially at IRZ and PRZ. 16S Microbiome profiling indicated that the maximum number of ASVs were from Bacteria, while only 6% accounted for Archaea, indicating a higher abundance of Bacteria from the sampled locations. This is similar to the results reported by Smedile et al. (2014), where they found that the 16S rRNA gene accounts for more than 70% of the water column indicating their predominance over the archaeal groups. A higher number of observed species and a higher Shannon index indicated that IRZ exhibited a higher microbial diversity than the other two locations, i.e., PRZ and BC20. This was also depicted by the higher number of phyla observed at IRZ (51 phyla) than at PRZ (44 phyla) and BC20 (37 phyla).

Proteobacteria were the most abundant microbial groups in the water column across the three locations. This could be because of the high metabolic diversity present among the Proteobacteria group. A higher abundance of Firmicutes, Bacteroidota and Actinobacteria was observed in the sediments, which could be due to the potential of these to form spores or their adaptability to extreme pressure and low temperatures. The hadal zones of the Japan Trench also showed a higher abundance of Bacteroidota and Actinobacteria (Hiraoka et al., 2020). Wenley et al. (2021) have reported a higher and consistent abundance of the Proteobacteria group at the Munida Microbial Observatory Time-Series in the Southern Ocean. Interestingly, the presence of the Cyanobacterial group was detected in the deeper regions water column (even the deep dark waters), i.e., below 600 m and in the sediment samples too. Wang et al. (2018) have obtained similar results, where they have detected the presence of Cyanobacteria from the sediment core samples from the Indian Ocean. Wenley et al. (2021) have also reported the presence of cyanobacterial communities from the deeper regions with less than 1% abundance. Poff et al. (2021) have reported the presence the photosynthetic materials from the bathypelagic zones and associated it with being attached to the sinking particles in the water column. Among Archaea, Thermoplasmatota was dominant in the water column, while the sediments were dominated by the Crenarchaeota group. A higher abundance of the organisms belonging to phylum Crenarchaeota has also been reported from the bathypelagic waters sampled from the Indian, Atlantic and Pacific Oceans during the Malaspina expedition (Ruiz-González et al., 2020).

The water column was found to be dominated by class Alphaproteobacteria, while the sediments were dominated by the Gammaproteobacteria group at all three sampling locations. Similar results were reported by Zinger et al. (2011), where Alphaproteobacteria was the dominant group in the pelagic communities, while Gammaproteobacteria was dominant in the benthic communities in the samples collected from the water column as well as the seafloor from the World’s oceans. However, it contrasts with the results obtained by Hoyningen-Huene et al. (2022), where a higher abundance of Alphaproteobacteria was detected from the sediment porewater from the Aldabra Atoll in Seychelles. Other abundant classes in the sediments include Bacilli, Actinobacteria and Acidimicrobiia. These are spore-forming organisms and their higher abundance in sediments could indicate their survival strategies in the deep-sea sediments. Some of the members of these classes are also known to perform redox reactions in the marine environment (Smedile et al., 2014). Among Archaea, Thermoplasmata was dominant in the water column, while Nitrososphaeria was dominant in the sediments.

Microbial genera, such as *Marinobacter*, *Marinimicrobia*_(SAR406_clasde), SAR11_clade, and *Rhodopirellula* were abundantly present in all the water samples throughout the column, and were found positively correlated with the nitrates and SPM. These genera are known to play an important role in carbon and nitrogen cycles (Strous et al., 2002). SAR11_clade has been found attached to the suspended particulate matter in the bathypelagic zones (Poff et al., 2021). Higher abundance in microbial diversity at genera level was detected in the lower depths of the water column in this study. A higher abundance of SAR11 clade has been reported as an indication of the degradation of planktonic-derived organic matter by bacteria (Han et al., 2022). The higher abundance of *Marinimicrobia* (SAR406_clade) and SAR202_clade at 200 and 600 m in the water column can be directly related to the negative correlation of the genera to oxygen and nitrate in the water samples. Hiraoka et al. (2020) have reported similar results where they detected a higher abundance of *Chloroflexi*, *Marinimicrobia* and *Woesearchaeota* with the decline in oxygen and nitrate concentrations from the sediment samples.

Microbial diversity in the sediments was found to decrease with an increase in depth from the seafloor. Similar results were obtained from the sediment porewater samples from the Aldabra Atoll, Seychelles where a decrease in phylogenetic diversity along with a shift in the microbial community was observed (Hoyningen-Huene et al., 2022). A higher abundance of Nitrosopumilaceae group of organisms can be related to their ammonia oxidizing potential in deep-sea sediments under aerobic conditions. Wang et al. (2018) have reported a higher abundance of *Nitrosopumilus* has been reported from the REY-rich sediment samples from the Indian Ocean. Similar results have been reported from the coastal sediments of Port Phillip Bay, Australia where they assessed the ammonia oxidation potential of *Nitrosopumilaceae* (Chen et al., 2021).

Microorganisms present in the deep-sea sediments are known to play a significant role in mediating the biogeochemical cycling of nutrients (Sogin et al., 2006), metal remineralization (Wang et al., 2018) and polymetallic nodule formation (Wu et al., 2013). Deciphering the microbial diversity in the deep sea and understanding their role in the biogeochemical cycles and mineralization of metals and nodule formation has become of strategic importance for many nations. There is still limited information on the microbial diversity from the deep sea of the Indian Ocean, and hence it is limiting our knowledge about their functional role in ecosystem functioning, nutrient transfer, different biogeochemical cycles and mineralization processes. This study provides an insight into the prokaryotic diversity present in the CIOB which can be used for future work. Further studies with the use of modern molecular tools such as metatranscriptomic and metaproteomics would deepen our understanding of the functional role of microbial communities in different oceanic processes.

## Conclusion

Microorganisms are known as key drivers for the major biogeochemical processes, and the knowledge of the diverse microorganisms in the deep sea is scarce and Indian Ocean is under-explored in terms of its microbial diversity and their role in different biogeochemical processes. However, with advancements in sequencing technologies, our understanding of deep-sea organisms is improving significantly. This data would be a substantial contribution to our understanding of the microbial diversity present in the depths of the CIOB and to the “Deep Ocean Mission,” which is a major initiative by the Government of India for exploration of the depths of Indian Ocean. The data reported here would contribute towards establishing a baseline of the existing microbial communities present there and can be used to assess any possible changes that could occur in the microbial community in case mining activities are initiated in the future in this region.

## Data availability statement

The datasets presented in this study can be found in online repositories. The names of the repository/repositories and accession number(s) can be found at: https://www.ncbi.nlm.nih.gov/genbank/, PRJNA668687.

## Author contributions

SS: Methodology, Validation, Writing – original draft, Formal analysis, Investigation, Visualization, Data curation. SD: Conceptualization, Funding acquisition, Methodology, Resources, Supervision, Validation, Writing – review & editing. MM-P: Writing – review & editing. JP: Formal analysis, Methodology, Writing – review & editing. SP: Formal analysis, Methodology, Writing – review & editing. SN: Formal analysis, Methodology, Writing – review & editing. AG: Formal analysis.
