# Supplementary material for: An insight into the prokaryotic diversity from a polymetallic nodule-rich region in the Central Indian Ocean Basin using next generation sequencing approach
Source: Front Microbiol. 2024 Mar 18;15:1295149. doi: 10.3389/fmicb.2024.1295149 (PMC10985493; doi:10.3389/fmicb.2024.1295149)

**Supplementary Figure 1.**


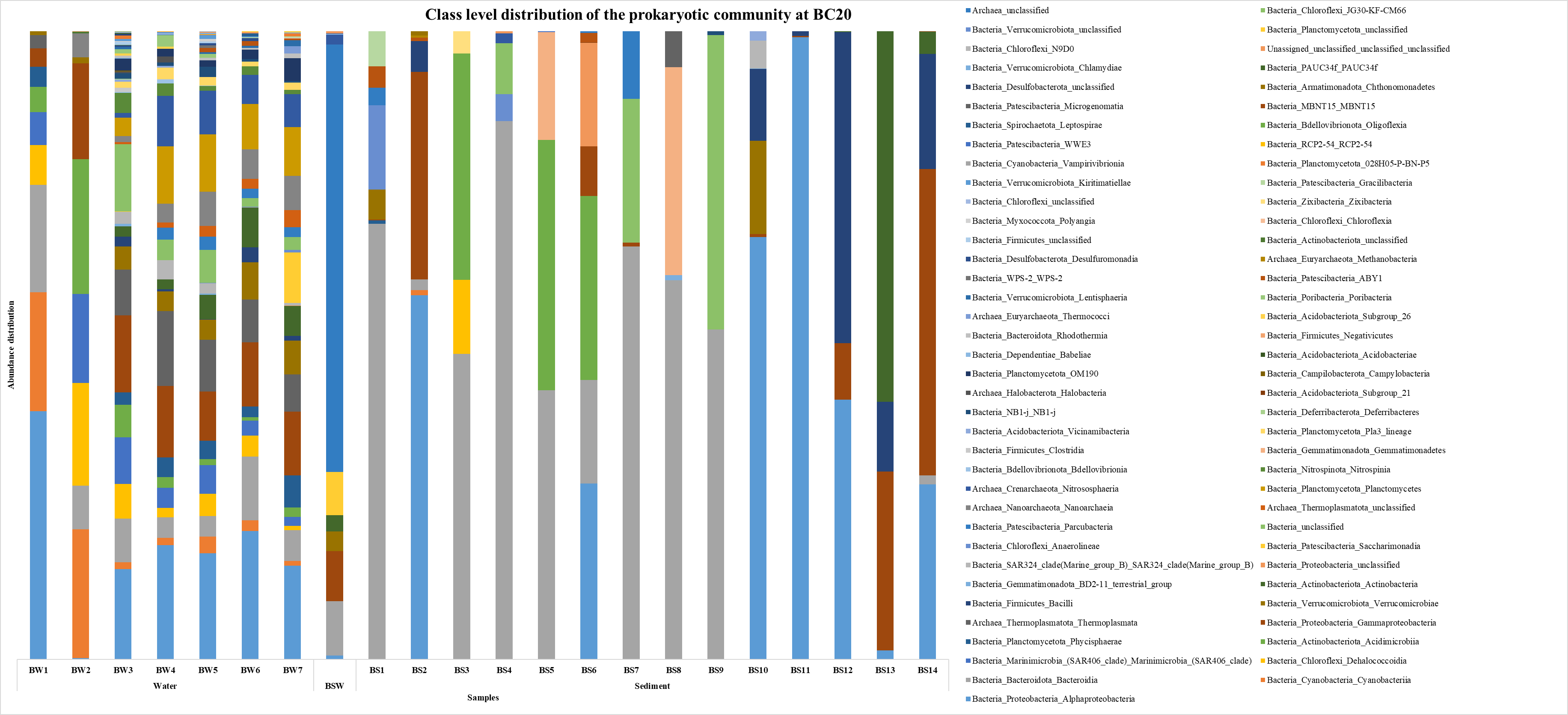


**Supplementary Figure 2.**


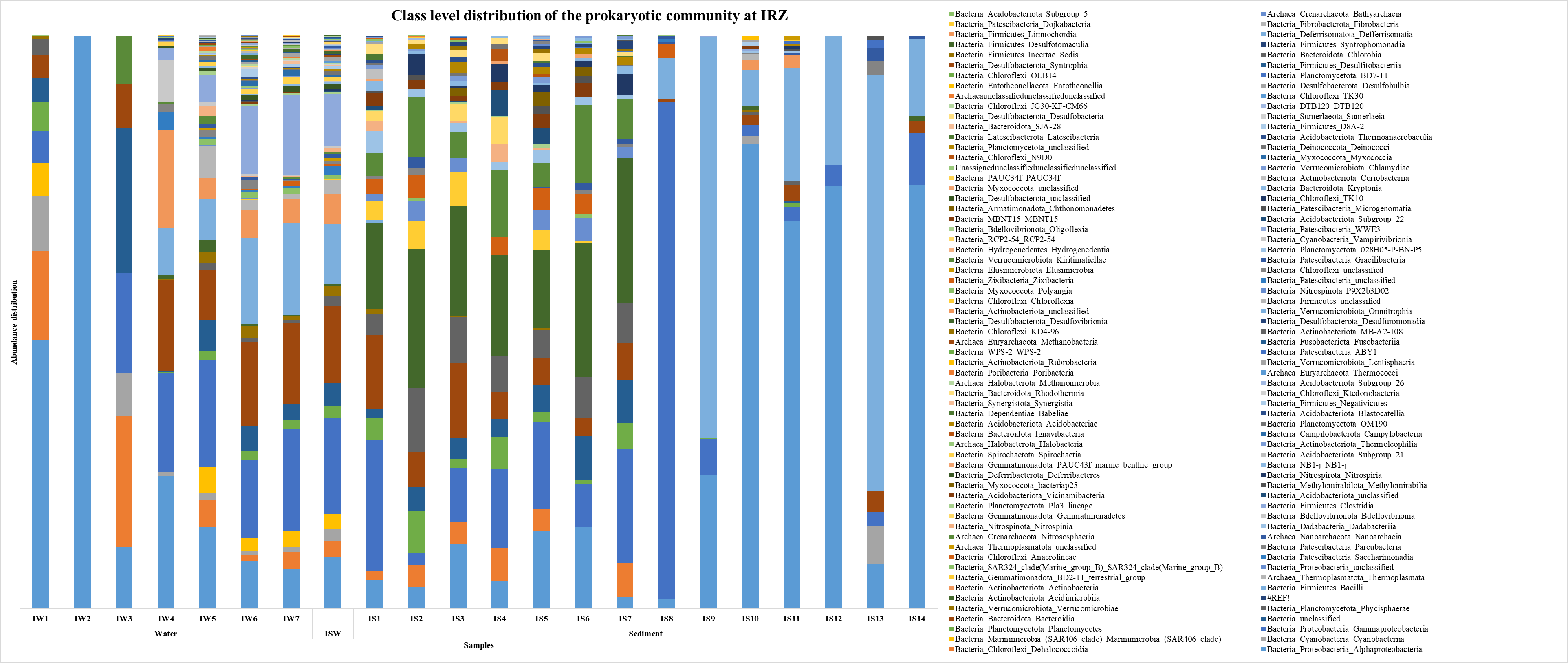


**Supplementary Figure 3.**


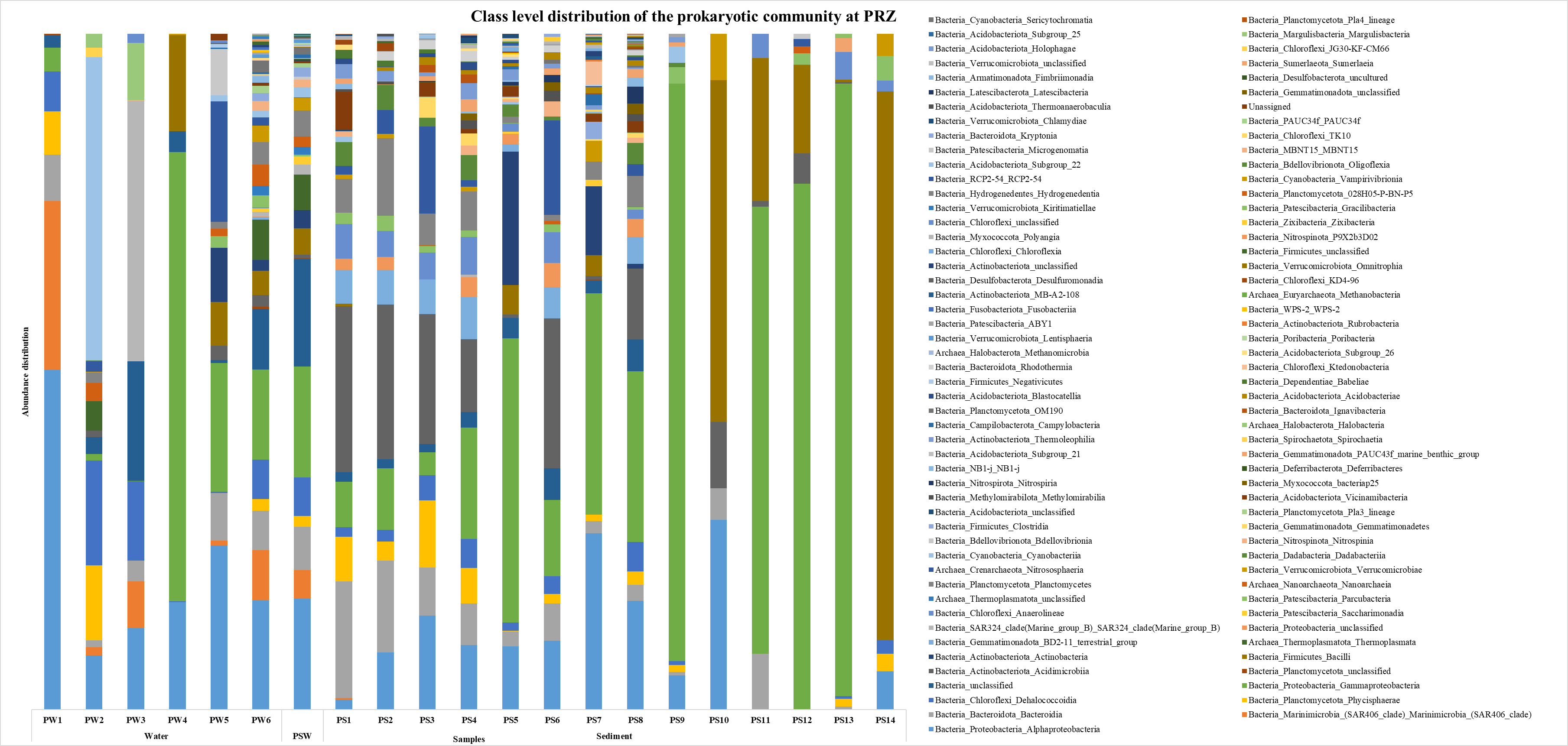


**Supplementary Figure 4:**


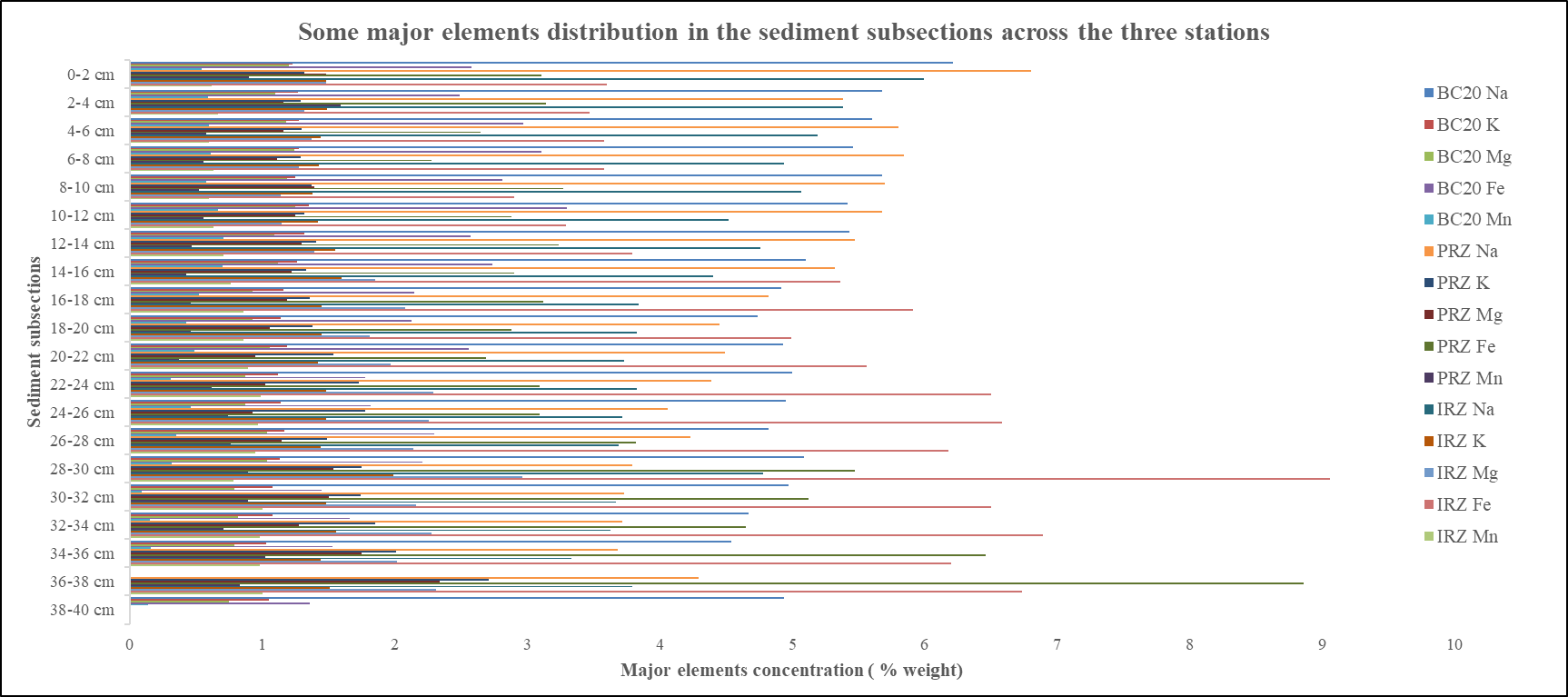


**Supplementary Figure 5:**


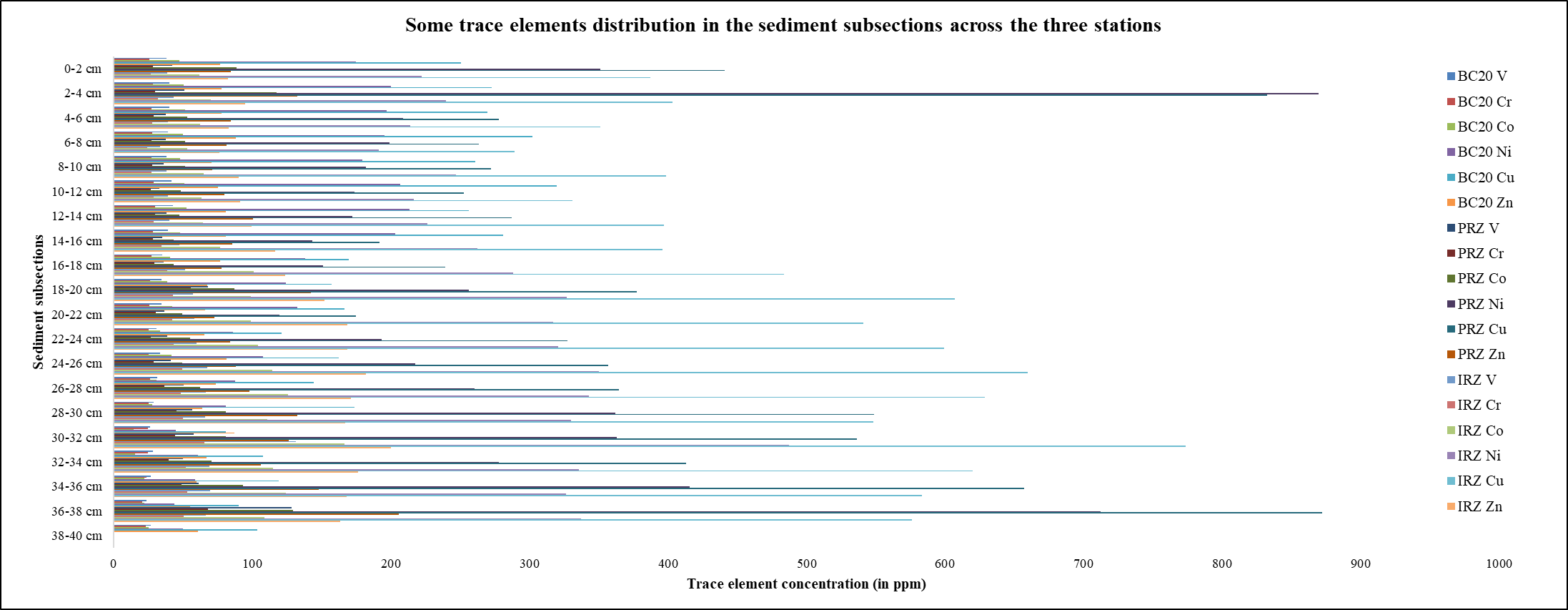

Supplement: Supplementary file 1 [file Data_Sheet_1.docx]
